# Validity of Ultrasound-Guided Identification of Dilated Lymphatic Vessels to Detect Early Breast Cancer-Related Lymphedema

**DOI:** 10.1055/a-2780-6493

**Published:** 2026-02-27

**Authors:** Akitatsu Hayashi, Takeo Fuziwara

**Affiliations:** 1 Department of Public Health, Institute of Science Tokyo, Tokyo, Japan; 2 Lymphedema Center, Department of Breast Center, Kameda General Hospital, Chiba, Japan

**Keywords:** lymphedema, BCRL, breast cancer, early diagnosis, screening, lymphedema–lymphatic physiology, reconstruction/lymphedema

## Abstract

**Background:**

Early detection of breast cancer-related lymphedema (BCRL) is critical for timely intervention and preventing progression, but diagnostic modalities are limited. We assessed the validity of ultrasound-guided identification of dilated lymphatic vessels (UIDL) to detect early BCRL.

**Methods:**

A retrospective, observational study with 300 female patients evaluated for suspected BCRL in a lymphedema center between April 2019 and March 2023. All patients underwent gold standard to detect BCRL, indocyanine green (ICG) lymphography, staged by the MD Anderson grading system, and UIDL from the proximal one-third of the upper arm to the distal one-third of the forearm circumferentially. The association between UIDL, with a cutoff of ≥0.5 mm in diameter, and the detection of early BCRL by ICG lymphography was evaluated.

**Results:**

In total, 264 (88%) cases were detected as BCRL by ICG lymphography. UIDL's area under the curve was 0.89 (95% CI: 0.84–0.94), with sensitivity of 86.2%, specificity of 91.7%, positive predictive value of 98.4%, and negative predictive value of 53.2%.

**Conclusion:**

UIDL showed high sensitivity and specificity in detecting early BCRL. This method may serve as a widely available, non-invasive screening tool during posttreatment surveillance.

## Introduction

Over the past several years, the incidence of breast cancer has risen markedly, with prevalence increasing progressively with advancing age. 1 Owing to continuous advances in multidisciplinary diagnostic and therapeutic strategies, patient survival rates have demonstrated a substantial upward trend, accompanied by notable improvements in prognosis and long-term outcomes. 2 There are various options for breast cancer patients. Most cases require surgical intervention for definitive diagnosis and treatment; however, such procedures carry the risk of postoperative complications, including infection, hematoma, cellulitis, seroma, and, most prominently, lymphedema resulting from disruption of the physiological integrity of the axillary lymphatic vessels. 3 4

Lymphedema represents a persistent and progressive complication of breast cancer surgery, particularly in patients undergoing axillary lymph node dissection. An estimated 10% to 56% of women who receive axillary lymph node dissection subsequently develop breast cancer-related lymphedema (BCRL), which is characterized by upper limb swelling, discomfort, functional impairment, and an elevated risk of cellulitis and recurrent hospitalizations. 5 6 7 8 9 BCRL is a chronic, progressive condition that is challenging to manage once established and can exert a profound, long-term detrimental effect on patients' quality of life. In addition to physical morbidity, affected individuals frequently experience psychological sequelae, including negative body image perception and significant emotional distress. 10 11 Therefore, BCRL focuses on prevention, early detection, and early intervention.

At present, the diagnostic assessment of BCRL comprises the acquisition of objective clinical measures, including bioelectrical impedance analysis, indocyanine green (ICG) lymphography, water displacement techniques, and limb circumference measurements, in conjunction with patient-reported subjective symptoms. 12 Based on subjective and objective data, researchers have developed many classification methods for lymphedema, including the MD Anderson classification of lymphedema using ICG lymphography ( Table 1 ). The MD Anderson grading system comprises six stages used to grade the degree of lymphedema severity from 0 to 5, where 0 is normal linear lymphatics with no dermal backflow. Stages 1 to 5 depict abnormal lymphatic patterns with various degrees of dermal backflow. 13 This staging system is widely used for BCRL staging and evaluation of surgical outcomes. However, most of these methods are not commonly used in most facilities, excluding specialized lymphedema institutions, because they need additional cost to purchase and contrast medium.

**Table 1:** Lymphedema classification based on indocyanine green-lymphography

Stages	MD Anderson staging
	Findings
Stage 0	No dermal backflow
Stage 1	Many patent lymphatics and minimal dermal backflow
Stage 2	Moderate number of patent lymphatics and segmental dermal backflow
Stage 3	Few patent lymphatics with extensive dermaI backflow
Stage 4	Dermal backflow involving the hand
Stage 5	ICG does not move proximally to the injection site

Abbreviation: ICG, indocyanine green.

**Fig. 1:**
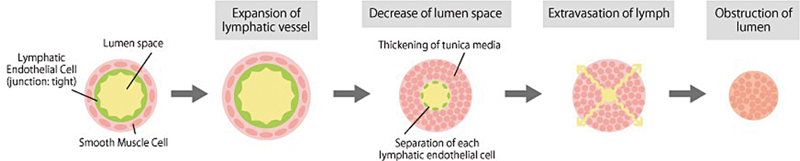
In the case of secondary lymphedema, the lymphatic vessels in extremities become sclerotic over time after lymph flow obstruction following expansion in early stage. They lose their function to drain lymph fluid along with thickening of tunica media and separation of each lymphatic endothelial cell step by step. Reproduced with permission by Kameda Medical Center. ( https://www.kameda.com/pr/lymphedema/en/post_8.html ).

Meanwhile, ultrasound, which is commonly used especially by breast surgeons, has recently been reported to be able to identify lymphatic vessels without contrast medium. 14 15 Mihara et al described the histological changes in collecting lymphatic vessels in cancer-related lymphedema patients. 16 They classified the lymphatic vessel into four different types: Normal, ectasis, contraction, and sclerosis, in which lymphatic vessels progressively show disassembly of focal adhesions, growth and transformation of smooth muscle cells, thickening of the basal membrane, and proliferation of collagen fibers ( Fig. 1 ). Ectasis type lymphatic vessels, which are seen in the early stages of lymphedema, are often dilated to 0.5 to 1 mm, making them relatively easy to identify with ultrasound compared to healthy individuals or later stages. 17 18 In this study, we used ultrasound to examine the presence or absence of lymphatic vessel dilation in patients who had breast cancer treatment and compared the results with the findings of ICG lymphography to determine whether ultrasound-guided identification of dilated lymphatic vessels (UIDL) is a useful screening examination for the early diagnosis of BCRL.

## Methods

The study included 300 female patients who visited our lymphedema center with suspected secondary upper limb lymphedema after breast cancer treatment (mastectomy, axillary lymph node dissection, sentinel node biopsy, chemotherapy, radiation therapy) between April 2019 and March 2023. Patient demographics are shown in Table 2 . All patients had ICG lymphography for diagnosis and UIDLs for checking the condition of lymphatic vessels. No patient had a potential allergic reaction to ICG. Lymphedematous volume was evaluated on the basis of the upper extremity lymphedema index, and the difference between the affected and healthy sides was determined. 19 This study was conducted under the institutional ethical review board. All patients provided written informed consent for participation in this retrospective observational study.

**Table 2:** Demographic characteristics

Characteristics	Value
Number of patients	300
Age, years	33–95 (52.2)
BMI, kg/m2	19.1–31.3 (23.7)
Postoperative period, months	2–86 (15.5)
ISL stage a	
0	166 (55.3%)
1	91 (30.3%)
2a	17 (5.7%)
2b	13 (4.3%)
3	13 (4.3%)
MD Anderson stage a	
0	36 (12%)
1	135 (45%)
2	75 (25%)
3	29 (9.7%)
4	9 (3%)
5	16 (5.3%)

Abbreviation: ISL, International Society of Lymphology.

Data are ranges (average), otherwise indicated.

a Data are counts (percentage).

### Indocyanine Green Lymphangiography

ICG lymphangiography was performed as follows: 0.2 mL of ICG (Diagnogreen 0.125%; Daiichi Pharmaceutical, Tokyo, Japan) was injected subcutaneously into the upper extremities at the first and fourth web spaces of the hand and the ulnar border of the palmaris longus tendon at the level of the wrist. 20 21 22 After approximately 60 minutes, fluorescent images of lymphatic drainage channels were obtained using a photodynamic eye infrared camera system (PDE; Hamamatsu Photonics K.K., Hamamatsu, Japan), which was used for the MD Anderson grading. In this study, stage 1 or 2 of this classification was defined as early BCRL.

### Detection of Lymphatic Vessel Using Ultrasound

The ultrasonographic findings from previous studies were used for the identification of the lymphatic vessels: Intermittent homogeneous and hypoechoic; no color seen with color Doppler mode; less likely to collapse with the transducer pressed to the skin and dilated in lymphedema, run beneath superficial fascia; and no convergence seen with arteries, venules, and nerves. 14 15 23 Based on this characteristic finding of the lymphatic vessels in ultrasound, UIDLs were performed from the proximal one-third of the upper arm to the distal one-third of the forearm circumferentially. To ensure that the identified vessels were not blood vessels, the vessels were checked with color Doppler mode. The quantity of dilated lymphatic vessels, which is identified by ultrasound, with a cutoff of ≥0.5 mm in diameter based on the previous studies, was documented. 17 18 Ultrasound was performed with the Vevo MD ultrasound device (FUJIFILM VisualSonics, Amsterdam, the Netherlands) using a 48-MHz linear array transducer. The pressure of the probe on the skin was minimized to avoid artificial deformation of the underlying structure. 15

### Statistical Analysis

For evaluating ultrasound as a screening examination for early BCRL, the findings of UIDLs were compared with ICG lymphangiography, and the sensitivity, specificity, positive predictive value (PPV), negative predictive value (NPV), and area under the curve (AUC) were calculated using Stata statistical software. A true positive is one or more dilated lymphatic vessels identified with ultrasonography, which were diagnosed as early stage (stage 1 or 2) on ICG lymphangiography. A false positive is more than one dilated lymphatic vessel identified with ultrasonography, which was not diagnosed as lymphedema (stage 0) on ICG lymphangiography. A true negative is any dilated lymphatic vessel identified with ultrasonography that was not diagnosed as lymphedema (stage 0) on ICG lymphangiography. A false negative is not any dilated lymphatic vessel identified with ultrasonography that was diagnosed as early stage (stage 1 or 2) on ICG lymphangiography.

## Results

**Fig. 2:**
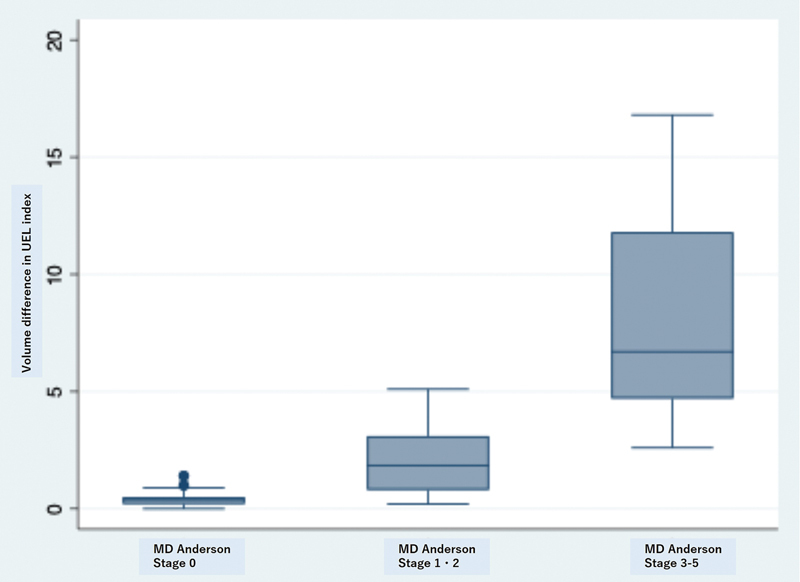
The volume difference in the upper extremity lymphedema index between the affected and healthy sides increases as the severity of lymphedema is advanced. Abbreviations: UEL, upper extremity lymphedema.

**Fig. 3:**
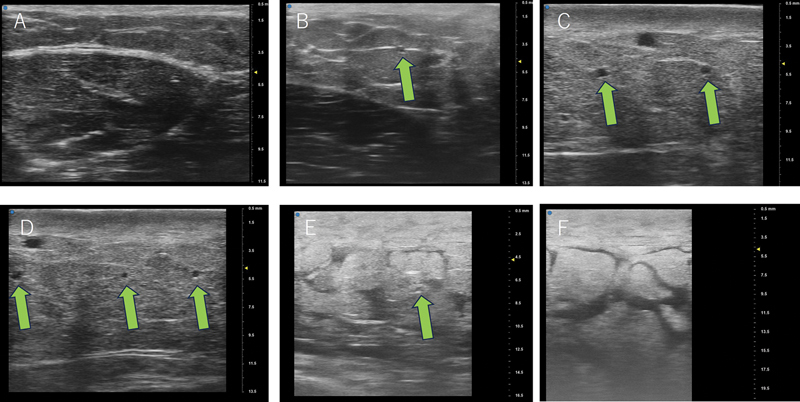
( **A** ) Ultrasound showed no dilated lymphatic vessels in MD Anderson stage 0 upper arm, ( **B** ) one dilated lymphatic vessel (green arrow) in stage 1 lymphedematous upper arm, ( **C** ) two dilated lymphatic vessels in stage 2, ( **D** ) three dilated lymphatic vessels in stage 3, ( **E** ) one dilated lymphatic vessels, fluid accumulation and moderate fat deposition in stage 4, and ( **F** ) no dilated lymphatic vessels, fluid accumulation, and severe fat deposition in stage 5.

The average age was 52.2 years (range, 33–95 years), and BMI was 23.7 (range, 19.1–31.3). ICG lymphatic staging based on the MD Anderson grading system demonstrated 36 patients with stage 0 lymphedema, 135 patients with stage 1, 75 patients with stage 2, 29 with stage 3, 9 with stage 4, and 16 patients with stage 5. The average volume difference between the affected and healthy sides is 0.4 in stage 0, 1.3 in stage 1, 3.1 in stage 2, 5.2 in stage 3, 10.9 in stage 4 and 12.7 in stage 5 ( Fig. 2 ). The average number of dilated lymphatic vessels is 0.2 in stage 0, 1.7 in stage 1, 4.2 in stage 2, 6.2 in stage 3, 1.7 in stage 4 and 0.2 in stage 5 ( Fig. 3 ). The number of true positives was 181, and that of false positives was 3. The number of true negatives was 33, and that of false negatives was 29 ( Table 3 ). Sensitivity, specificity, PPV, NPV, and AUC of the UIDLs to detect early BCRL were 86.2%, 91.7%, 98.4%, 53.2%, and 0.89 (95% CI: 0.84–0.94), respectively.

**Table 3:** Comparison between ultrasound and indocyanine green-lymphography (MD Anderson stage 0–2)

		ICG-L	
		(+) a	(−) b
US	(+) c	181	3
	(−) d	29	33

Abbreviations: ICG-L, indocyanine green-lymphography; US, ultrasonography.

a “ + ” denotes a state where diagnosed as an early stage of lymphedema with ICG-L.

b “ − ” denotes a state where not diagnosed as lymphedema with ICG-L.

c “ + ” denotes a state where more than one dilated lymphatic vessel is identified with US.

d “ − ” denotes a state where no dilated lymphatic vessel is identified with US.

## Discussion

Due to different surgical styles and axillary lymph node management, patients in the early postoperative period may exhibit differing degrees of symptoms associated with scar tissue traction (e.g., tightness and stiffness), manifestations attributable to intercostal brachial nerve injury, and a constellation of symptoms related to surgical trauma, including pain and localized tenderness. 24 This is like the symptoms of BCRL, but does not provide a diagnosis of lymphedema on this basis. That is why early-stage BCRL is easily overlooked in breast cancer posttreatment follow-up outpatient clinics. Accumulating evidence indicates that surveillance and early detection strategies are more cost-effective than deferring intervention until the onset of clinical symptoms or overt swelling. 25 It is noteworthy that a substantial proportion of patients with clinically apparent lymphedema do not report corresponding subjective symptoms; therefore, cancer survivors, even if they do not report any symptoms, should undergo screening for lymphedema. 26 27

There is often wide individual variation in the complaints and clinical presentation of patients with BCRL. Therefore, there is no “gold standard” for the early diagnosis of BCRL; the specificity of subjective perception and self-reported measures is limited, rendering symptom-based indicators alone insufficient for the identification of patients with early-stage lymphedema. To improve the accuracy and precision of diagnosis, it has been necessary to combine limb circumferential measurements, bioelectrical impedance, and even imaging methods to improve the accuracy and precision of diagnosis.

The diagnosis is often based on the difference in arm circumference between the two upper limbs. However, some patients have subjective edema symptoms before the limb circumference changes, suggesting the presence of early-stage lymphedema. Therefore, it is difficult to diagnose lymphedema by measuring arm circumference. On the other hand, it is reported that bioelectrical impedance analysis evaluates body tissue composition by applying an electrical current (5–1,000 kHz) and monitoring impedance responses, enabling detection prior to the onset of overt limb changes or subjective symptoms. Ultimately, measurements of extracellular fluid composition are utilized to determine the presence of lymphedema and to assess the severity of edema. 28 Recent study showed that bioelectrical impedance monitoring has a higher sensitivity for diagnosing lymphedema compared to subjective symptom questionnaires. 29 Therefore, combining the symptom index scale with limb circumference measurement with bioelectrical impedance can improve the diagnostic accuracy of BCRL. In addition, lymphatic radionuclide imaging or ICG fluorescence imaging is also the gold standard for the detection of early-stage lymphedema, especially for reconstructive microsurgeons, because these modalities are useful not only for diagnosis but also for preoperative planning of surgery for lymphedema. 13 On the other hand, most diagnosis and breast cancer treatment institutions do not have these kinds of professional equipment and imaging modalities.

A recent study revealed that a preoperative ultrasound detection technique without contrast medium enabled surgeons to dissect larger-diameter lymphatic vessels and resulted in a significant decrease in lymphedema index. 23 The reason is that the lymphatic vessels responsible for lymphedema are blocked proximally, so lymphatic vessels distal to the point of blockage are dilated. The purpose of this study was to examine the validity of UIDLs in the early diagnosis of BCRL by examining the presence or absence of dilated lymphatic vessels using ultrasound, a non-invasive examination that is available at many facilities and does not require contrast agents, and comparing it with ICG lymphangiography staged by the MD Anderson grading system. The results showed high sensitivity, specificity, and AUC of this technique.

In this study, the number of dilated lymphatic vessels increased to a moderate stage (MD Anderson stage 3) but then decreased as the severity increased. This result was consistent with the results of previous pathological studies, which showed that lymphatic vessels become sclerotic over time after lymph flow obstruction and lose their function to drain lymph flow in peripheral lymphedema patients. 16 Furthermore, the volume difference between the affected and healthy sides increases as severity is advanced, suggesting that when the number of dilated lymphatic vessels is low, the severity can be determined by the difference between the affected and healthy sides in lymphedema. In other words, if there is little difference between the affected and healthy sides but one or more dilated lymphatic vessels are observed, early stages of lymphedema should be suspected.

On the other hand, although lymphatic vessel dilation was observed in stage 0 cases, this may indicate that lymphatic vessel stenosis, even before lymph leakage due to lymphatic vessel obstruction is observed, and in such cases, it may be possible to prevent progression by performing lymphatic drainage from an early stage.

There are several limitations in the present study. First, the acquisition of an accurate ultrasonographic image was highly operator-dependent and machine type-dependent. Because our ultrasound scanning of the lymphatic vessels was performed by one examiner and one machine type, interoperator and intermachine reliability could not be evaluated. Further studies are required to investigate the learning curve of performing ultrasound and compare it in different operators and machine types. Second, the high PPV and NPV in this study may be dependent on the prevalence of the target population. Because the subjects of this study were limited to patients undergoing a diagnosis of BCRL. Further studies are required to examine the validity of this technique in a more general population. Third, lymphoscintigraphy is still required for the final definitive diagnosis of lymphedema, but it was not used in this study. Future studies comparing lymphoscintigraphy and ultrasound are needed. Fourth, long-term follow-up studies are required to determine the clinical significance and natural course of the observed lymphatic changes.

In recent years, with the increasing number of breast cancer patients and the improving outcomes of cancer treatment, the number of patients with BCRL is expected to increase even more than before. In clinical practice, breast surgeons often refer patients to lymphedema specialists or therapists when they notice asymmetry between the affected and healthy upper limbs during physical examination. Because treatment is initiated only after lymphedema has progressed to a certain extent, the effectiveness of treatment tends to be limited. This study suggests that UIDL, a non-invasive examination familiar to not only reconstructive microsurgeons but also breast surgeons and medical technologists, is useful not only for the decision of surgery for lymphedema but also for early diagnosis of BCRL. Regular use of ultrasound during follow-up after breast cancer treatment, combined with the patient's subjective symptoms, would enable easier and faster screening and early diagnosis of BCRL, leading to earlier treatment and potentially preventing lymphedema progression.

### Conclusion

UIDL is a feasible, non-invasive method for detecting early BCRL. In this retrospective cohort of 300 patients, this technique demonstrated high sensitivity, specificity, and AUC for ICG-defined early-stage lymphedema (MD Anderson stages 1–2). Because ultrasound is widely available in breast clinics and requires no contrast medium, routine incorporation of this technique into posttreatment surveillance could facilitate earlier identification and referral for conservative or surgical interventions, potentially limiting progression and improving patient outcomes. Future multicenter and longitudinal studies should validate diagnostic thresholds, assess the impact on clinical management and outcomes, and define standardized scanning protocols to optimize ultrasound use as a screening tool for early BCRL.
